# Artificial Intelligence Applied to Chest X-ray for Differential Diagnosis of COVID-19 Pneumonia

**DOI:** 10.3390/diagnostics11030530

**Published:** 2021-03-16

**Authors:** Christian Salvatore, Matteo Interlenghi, Caterina B. Monti, Davide Ippolito, Davide Capra, Andrea Cozzi, Simone Schiaffino, Annalisa Polidori, Davide Gandola, Marco Alì, Isabella Castiglioni, Cristina Messa, Francesco Sardanelli

**Affiliations:** 1 Department of Science, Technology, and Society, Scuola Universitaria IUSS, Istituto Universitario di Studi Superiori, Piazza della Vittoria 15, 27100 Pavia, Italy; salvatore@deeptracetech.com; 2 DeepTrace Technologies S.R.L., via Conservatorio 17, 20122 Milano, Italy; interlenghi@deeptracetech.com (M.I.); polidori@deeptracetech.com (A.P.); 3 Department of Biomedical Sciences for Health, Università degli Studi di Milano, Via Mangiagalli 31, 20133 Milano, Italy; caterina.monti@unimi.it (C.B.M.); davide.capra@unimi.it (D.C.); andrea.cozzi1@unimi.it (A.C.); francesco.sardanelli@unimi.it (F.S.); 4 Department of Radiology, ASST Monza—Ospedale San Gerardo, Via Pergolesi 33, 20900 Monza, Italy; davide.ippolito@unimib.it (D.I.); d.gandola1@campus.unimib.it (D.G.); 5 Unit of Radiology, IRCCS Policlinico San Donato, Via Morandi 30, 20097 San Donato Milanese, Italy; schiaffino.simone@gmail.com; 6 Department of Diagnostic Imaging and Stereotactic Radiosurgery, C.D.I. Centro Diagnostico Italiano S.p.A., Via Saint Bon 20, 20147 Milano, Italy; marco.ali@cdi.it; 7 Department of Physics, Università degli Studi di Milano-Bicocca, Piazza della Scienza 3, 20126 Milano, Italy; 8 Institute of Biomedical Imaging and Physiology, Consiglio Nazionale delle Ricerche, Via Fratelli Cervi 93, 20090 Segrate, Italy; 9 School of Medicine and Surgery, Università degli Studi di Milano-Bicocca, Piazza dell’Ateneo Nuovo 1, 20126 Milano, Italy; cristina.messa@unimib.it; 10 Fondazione Tecnomed, Università degli Studi di Milano-Bicocca, Palazzina Ciclotrone—Via Pergolesi 33, 20900 Monza, Italy

**Keywords:** artificial intelligence, neural networks, SARS-CoV-2, COVID-19, community-acquired pneumonia, chest X-ray, sensitivity, specificity, differential diagnosis

## Abstract

We assessed the role of artificial intelligence applied to chest X-rays (CXRs) in supporting the diagnosis of COVID-19. We trained and cross-validated a model with an ensemble of 10 convolutional neural networks with CXRs of 98 COVID-19 patients, 88 community-acquired pneumonia (CAP) patients, and 98 subjects without either COVID-19 or CAP, collected in two Italian hospitals. The system was tested on two independent cohorts, namely, 148 patients (COVID-19, CAP, or negative) collected by one of the two hospitals (independent testing I) and 820 COVID-19 patients collected by a multicenter study (independent testing II). On the training and cross-validation dataset, sensitivity, specificity, and area under the curve (AUC) were 0.91, 0.87, and 0.93 for COVID-19 versus negative subjects, 0.85, 0.82, and 0.94 for COVID-19 versus CAP. On the independent testing I, sensitivity, specificity, and AUC were 0.98, 0.88, and 0.98 for COVID-19 versus negative subjects, 0.97, 0.96, and 0.98 for COVID-19 versus CAP. On the independent testing II, the system correctly diagnosed 652 COVID-19 patients versus negative subjects (0.80 sensitivity) and correctly differentiated 674 COVID-19 versus CAP patients (0.82 sensitivity). This system appears promising for the diagnosis and differential diagnosis of COVID-19, showing its potential as a second opinion tool in conditions of the variable prevalence of different types of infectious pneumonia.

## 1. Introduction

Differential diagnosis of COVID-19 from other types of pneumonia has been a high-priority research topic and clinical aim since the early stages of the current pandemic [1,2]. Prompt identification of COVID-19 cases is paramount to ensure proper management and better patient outcomes [3,4,5]. Moreover, any tool to be applied for this aim should have a good cost–benefit ratio for the healthcare service, be able to adapt to heterogeneous settings, and be also useful outside COVID-19 pandemic peak, enabling accurate differential diagnosis with other types of pneumonia, such as non-COVID-19 community-acquired pneumonia (CAP) [2,5,6,7,8].

The current reference standard for the detection of COVID-19 is the detection of SARS-CoV-2 by reverse transcription polymerase chain reaction (RT-PCR) [3,9]. However, due to intrinsic shortcomings of this diagnostic modality and to the high prevalence and clinical impact of COVID-19, chest imaging has been widely used to triage suspect cases [10,11,12]. A meta-analysis on the diagnostic performance of computed tomography (CT) showed a 94% pooled sensitivity, specificity being however under 40% [13]. Moreover, the use of CT implies higher healthcare costs since CT scanners have relatively limited availability, even in high-income countries, and CT equipment and rooms need sanitization after each use involving suspected or confirmed cases unless a continuous series of confirmed cases has to be studied [14,15,16]. In this context, the use of chest X-ray imaging (CXR) has become increasingly commonplace to evaluate patients presenting with symptoms potentially associated with COVID-19 such as fever, cough, or dyspnea [17,18,19,20,21]. Typical COVID-19 abnormal findings reported at CXR are portions of the lungs appearing as a “hazy” shade of grey instead of normal well-aerated parenchyma, representing pneumonia foci, with fine linear structures representing blood vessels [18], the so-called ground-glass opacities, which are also well-detected at CT [22]. Since these findings are among the first radiological manifestations of COVID-19 pneumonia, it could be hypothesized that an accurate CXR reading could aid the early diagnosis of COVID-19 pneumonia, also providing the additional benefit of differential diagnosis from CAP.

Recently, artificial intelligence (AI) and deep learning, in particular convolutional neural networks (CNNs), have been proven an effective and reliable tool to both automate and improve diagnosis and prognosis of various diseases, including pneumonia, as shown by competitors at the 2018 Kaggle Challenge for Chest X-ray images [23]. Furthermore, AI approaches have shown potential in performing differential diagnoses between different types of pneumonia, namely bacterial and viral CAP [24,25]. Early in the pandemic, AI was employed in COVID-19 diagnosis by various teams, showing sensitivities and specificities well over 0.90 by both machine learning [26,27,28] and deep learning [29,30,31,32,33,34,35,36,37,38,39,40] techniques. As already pointed out by Farhat et al. [41], Shi et al. [42], and López-Cabrera et al. [43], pre-trained CNN-based systems emerge as the most popular and powerful approaches for the automatic classification of images of suspected COVID-19 patients. However, most of the published studies so far have focused on CT, only some of them using CXR [26,28,31,36,40]. Moreover, only the studies by Kana et al. [26] and Ucar et al. [40] demonstrated the applicability of their models also to the differential diagnosis of COVID-19 versus other types of pneumonia. Specifically, Kana et al. [26] implemented a transfer learning model based on CXRs to differentiate healthy individuals, bacterial or viral pneumonia versus COVID-19 pneumonia, obtaining near-100% accuracy. Ucar et al. [40] fine-tuned a SqueezeNet using a Bayesian optimization approach, reaching 98% accuracy in classifying normal subjects, patients with non-COVID-19 CAP and COVID-19 patients. However, all the aforementioned studies did not use an independent testing set (neither temporally nor spatially independent) that would allow for an unbiased evaluation of model performance.

The aim of our study was to evaluate the two-sided role of AI applied to CXR in patients suspected to be affected by COVID-19 pneumonia, i.e., outright COVID-19 diagnosis and differential diagnosis from other CAPs. The general purpose was to present an effective tool supporting the diagnosis of COVID-19 pneumonia in the perspective of offering a second opinion to radiologists or a preliminary assessment when a radiologist is not immediately available. With these aims, taking into consideration the strengths and limitations of the current literature, which points to a consolidated superior performance of CNNs for image classification tasks [44], we trained and cross-validated a ResNet-50 architecture. The model was applied to CXRs for supporting the differential diagnosis of COVID-19. Model performance was evaluated using two independent testing sets.

## 2. Materials and Methods

### 2.1. Study Protocol

The local Ethics Committee approved this retrospective study on 8 April 2020, and informed consent was waived due to the retrospective nature of the study.

### 2.2. Population and Datasets

#### 2.2.1. Training, Validation, and First Independent Testing

For the training/validation phase and the first independent testing (independent testing I) consecutive patients referred to the emergency department (ED) of two hospitals in Lombardy, Italy (IRCCS Policlinico San Donato, San Donato Milanese, Center 1; ASST Monza—Ospedale San Gerardo, Monza, Center 2) were included in the study. From these centers, two groups of patients were assessed according to two different timeframes—the first group referred to EDs between mid-February 2020 and mid-March 2020 and the second in the same period during 2019.

The first group included patients with RT-PCR-confirmed SARS-CoV-2 infection undergoing CXR on ED admission. Digital CXR was performed in two projections (posteroanterior and lateral) in the radiology unit or in one anteroposterior projection at bedside in the ED. Whenever both the posteroanterior and lateral projections were available, only the former was included for further analysis.

In the second group, we included patients with suspected CAP undergoing CXR on ED admission. As for patients of the first group, digital CXR was performed in two projections (posteroanterior and lateral) in the radiology unit or in one anteroposterior projection at bedside in the ED. Again, only the posteroanterior projection was considered when two projections were available.

Radiological labels for both groups were attributed by two radiologists of the two centers involved in this study (with 8 years and 13 years of experience in CXR interpretation).

#### 2.2.2. Second Independent Testing

For the external testing (independent testing II) of our AI model, a third group of patients was retrieved from the publicly available dataset “AIforCOVID” [45]. Ethics Committee approval was obtained also for this study. The AIforCOVID dataset, collected between March 2020 and June 2020, includes posteroanterior CXRs of RT-PCR-confirmed COVID-19 patients from six other hospitals in Italy. Patients from this dataset are categorized as having “mild” or “severe” disease according to their clinical outcome—patients assigned to the “mild” group were either sent to domiciliary isolation or were hospitalized in ordinary wards without the need of ventilatory support, while the “severe” group included all hospitalized patients that required ventilation support, intensive care, and/or died during hospitalization.

### 2.3. AI System

The TRACE4© radiomic platform (DeepTrace Technologies S.R.L., Milano, Italy) [46] was used to classify CXRs of the different groups of patients. This platform allows training, validation, and testing of different AI systems combined with different feature-extraction methods applied to medical images for classification purposes.

The TRACE4© platform includes full workflow for radiomic analysis (i.e., compliant to the guidelines of International Biomarker Standardization Initiative [47]); different feature extraction and selection methods, and different ensembles of machine-learning techniques such as support vector machines, random forests, deep learning and transfer learning of neural networks.

The classification tasks of interest were binary (COVID-19 versus negative, COVID-19 versus CAP), considering the following cases: COVID-19, all patients with positive RT-PCR; negative, all patients from the second group without any CXR finding; and CAP, all patients from the second group with positive CXR findings for CAP. The deep-neural-network classifier proposed in this work was implemented on the ResNet-50 architecture, i.e., a convolutional neural network composed of 50 layers. The network is able to learn a rich feature representation of the input classes (more than a million images from the ImageNet database [48]). To train and test an ensemble of 10 convolutional neural networks (based on the ResNet-50 architecture), a 10-fold cross-validation procedure was used. The classification outputs of each of the 10 models concurring to the ensemble were then merged by sum rule (ensemble-averaging of class probability) to obtain the final classification output of the ensemble of classifiers.

This feature representation is then used to classify new samples (new images, in this case) to one of the input classes. In order to specialize the pre-trained ResNet-50 network to the binary-discrimination tasks of interest in our study (i.e., COVID-19 versus negative and COVID-19 versus CAP), we applied a fine-tuning process to the last layers of the original ResNet-50 architecture (COVID-19 versus negative, COVID-19 versus CAP) [48].

CXR images were resized to the input size of the architecture (i.e., 224 pixels by 224 pixels) before being fed into the deep neural network. Automatic data-augmentation techniques were applied to the resized CXR set during the training of the classifier—this operation, which includes image rotation, shear, and reflection, aims at increasing CXR image diversity among different training phases (epochs), thus increasing the performance of the training procedure. No further data processing was applied to the CXR images used as input to the deep-learning network nor during the training-and-classification process.

To obtain further estimates of the performance of our AI system, the model resulting from the training procedure was tested on the two independent sets of patients (independent testing I and II); none of these two sets included patients from the cross-validation procedure.

Performance metrics for both cross-validation and independent testing I are presented in terms of sensitivity, specificity, and areas under the receiver operating curve (ROC AUC), with their 95% confidence intervals (95% CIs) reported only for cross-validation performances. For independent testing II, since no negative controls or CAP patients are present in this independent cohort, sensitivity only was calculated. In this second independent testing, subgroup analysis according to COVID-19 severity (“mild” or “severe” group) was also performed.

## 3. Results

**Figure 1:**
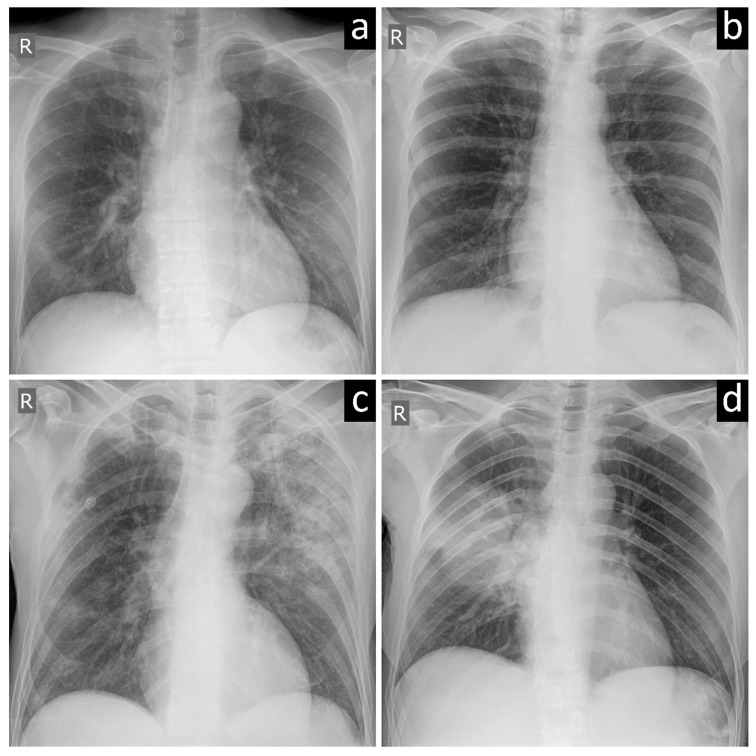
Chest X-ray images of subjects with (**a**) COVID-19 pneumonia, (**b**) negative examination, (**c**) viral pneumonia, and (**d**) bacterial pneumonia.

A total of 162 patients who underwent CXR and tested positive for SARS-CoV-2 infection at RT-PCR in Centers 1 and 2 from 21 February 2020 to 16 March 2020 were included in our retrospective evaluation (first group of patients). From all patients admitted to the two hospitals roughly in the same period the year before, 112 patients with CAP and 158 negative controls were included, accounting for a total of 270 patients in the second group. For the third group, 820 patients RT-PCR-confirmed COVID-19 patients were retrieved for our retrospective evaluation from the AIforCOVID database, 384/820 (47%) being categorized in the “mild” subgroup of this database, 436/820 (53%) in the “severe” subgroup. Table 1 shows the distribution of the 1252-included patients, while Figure 1 shows examples of CXRs with typical COVID-19 pneumonia (Figure 1a), negative findings (Figure 1b), and non-COVID-19 viral and bacterial pneumonia (Figure 1c,d).

We trained and validated (cross-validated) the proposed AI system on CXRs of 284/432 (66%) patients from Centers 1 and 2, as follows: 98 COVID-19 patients (48 from Center 1, 50 from Center 2) versus 98 negative subjects (48 from Center 1, 50 from Center 2), with a 0.91 sensitivity (95% CI 0.85–0.97), 0.87 specificity (95% CI 0.82–0.92), and area under the curve (AUC) of 0.93 (95% CI 0.88–0.98); 98 COVID-19 patients (48 from Center 1, 50 from Center 2) versus 88 CAP patients (42 from Center 1, 46 from Center 2) with a 0.85 sensitivity (95% CI 0.76–0.94), 0.82 specificity (95% CI 0.77–0.87), and 0.94 AUC (95% CI 0.90–0.98).

Independent testing I was conducted on the remaining CXRs of 148/432 (34%) patients from Center 2 (independent testing I), as follows: 64 COVID-19 versus 60 negative subjects with current sensitivity, specificity, and AUC of 0.98, 0.88, and 0.98, respectively; 64 COVID-19 versus 24 CAP patients, with current sensitivity, specificity, and AUC of 0.97, 0.96, and 0.98, respectively.

Independent testing II conducted on the CXRs of the 820 COVID-19 patients from the AIforCOVID dataset (third group of patients) showed 652 COVID-19 patients correctly classified versus negative subjects (with a sensitivity of 0.80) and 674 COVID-19 patients correctly classified versus CAP (with sensitivity of 0.82). Subgroup analysis on the “mild” and “severe” COVID-19 patients showed that the proposed AI system for the discrimination between COVID-19 and negative subjects correctly classified 264 out of 384 patients in the “mild” subgroup (sensitivity 0.69) and 388 out of 436 patients in the “severe” subgroup (sensitivity 0.89). Conversely, the proposed AI system for the discrimination between COVID-19 and CAP patients correctly classified 284 out of 384 patients in the “mild” subgroup (sensitivity 0.74) and 390 out of 436 patients in the “severe” subgroup (sensitivity 0.89).

**Figure 2:**
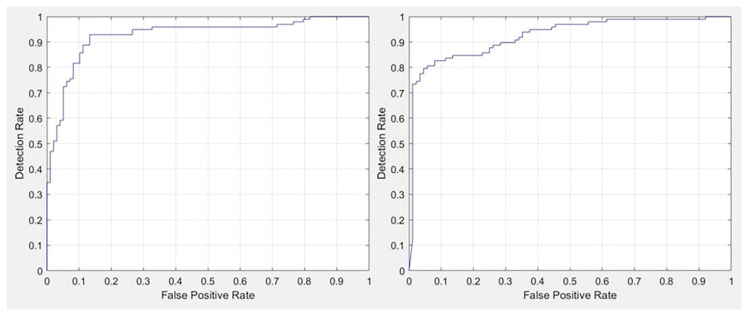
(**Left**) areas under the curve at receiver operating characteristic analysis for COVID-19 versus negative and (**Right**) for COVID-19 versus community-acquired pneumonia in the cross-validation phase.

Table 2, Table 3 and Table 4 detail the performance of the proposed AI system, with corresponding receiver operating curves (ROCs) shown in Figure 2.

## 4. Discussion

Over the last year, the COVID-19 pandemic showed an ever-shifting time- and space-related epidemiological profile worldwide [49,50]. However, in all phases of pandemic waves, quick and accurate diagnosis remains of utmost importance [51,52], in particular when facing viral variants [53,54] and the need to take advantage of the effect of vaccination campaigns [54,55]. In this context, CXR has emerged as a crucial first-line diagnostic tool for the detection of COVID-19 pneumonia in the ED setting [18] and beyond [56,57], given its high availability, low associated costs, and accuracy in pneumonia diagnosis [7,10,18,58,59].

Although COVID-19 pneumonia appears on CXR with characteristic features, many of them are also shared by other viral types of pneumonia [7,18,60]. The improvement of CXR diagnostic performance would be paramount to ameliorate decision making regarding patient management, which strongly relies on the initial assessment, considering both the intrinsic shortcomings of RT-PCR testing and the difficulties of implementing a CT strategy for early diagnosis [10,14,15,16].

For the purpose of diagnosis, we trained and tested an ensemble of ten convolutional neural networks with CXRs of 98 COVID-19 patients referring to the EDs of two university hospitals in northern Italy (Center 1 and Center 2) during the first 2020 pandemic wave in northern Italy and 98 negative subjects from approximately the same period of 2019. We then tested the proposed AI system on an independent cohort of 148 patients not used during training coming from one of these two centers (independent testing I). The AI model was able to automatically classify COVID-19 and negative subjects with a sensitivity of 0.98, a 0.88 specificity, and a 0.94 AUC. Furthermore, another independent testing (independent testing II) on a public dataset of 820 COVID-19 patients showed good generalization abilities of our AI tool, yielding an average sensitivity of 0.80 for the task of diagnosis versus negative subjects, with even higher performance when considering COVID-19 patients with severe disease, as expected.

For the purpose of differential diagnosis, we trained and tested the ensemble of convolutional neural networks with CXRs of 98 COVID-19 patients and 88 patients with CAP (collected in 2019) from Center 1 and Center 2. The temporal selection criterion for CAP patients was enforced to ensure that no patient could carry undetected SARS-CoV-2 infection. In independent testing I, this AI model was able to classify COVID-19 and CAP patients with a 0.97 sensitivity, a 0.96 specificity, and a 0.94 AUC. Of note, independent testing II confirmed the good generalization abilities of our AI tool, performing with an average sensitivity of 0.82 for the task of COVID-19 versus CAP patients, with better performance for COVID-19 patients with severe disease, as expected again.

The presented AI tool yielded an interesting performance for the detection of COVID-19 compared to negative subjects and for the differential diagnosis with CAP. These performances open promising perspectives for our AI system to be used in clinical practice, thanks to a relatively high sensitivity (ranging 0.80–0.98 in the independent testing) with an interesting specificity (0.88). Especially in conditions of variable prevalence [50,61,62], due to local viral waves, effects of vaccination, and the appearance of viral variants [54,55], the availability of an AI tool as a second opinion support system may be useful to increase the diagnostic performance. We imagine a practical possibility of combining human and AI reading according to the rule of double reading. When one reading (human or AI) is positive and the other one is negative, in the case of high prevalence, the overall result will be given as positive; hence, maximizing sensitivity and negative predictive value. Conversely, in the same combination of contradictory results but in a low prevalence setting, a third (human) reader will be asked to take the decision, trying to maximize specificity and positive predictive value. The next challenge will be to appraise how the human grasp on different settings, for instance, a pandemic context versus relative normalcy, may interact with the performance of our AI tool [63].

Our research has some important limitations.

First, concerning the performance achieved by our AI system, in this study, we report the sensitivity, specificity, and AUC obtained by training the network with only 98 COVID-19 patients. Overall, our system was able to detect both COVID-19 subjects (sensitivity ranging 0.80–0.97) and non-infected subjects (negative and CAP, specificity 0.88) with high and quite balanced performance. Despite this, our AI system could improve these performances when trained with more CXRs in a larger multicenter setting. Even though a two-center CXRs set was used to train and validate the AI system (cross-validation), the first test set of CXRs (independent testing I) was separated from the set used to train and validate the AI but came from one of these centers, while the second test set of CXRs (independent testing II) came from different centers and also timeframe with respect to the training dataset.

Second, our AI model is currently not able to define the stage or to predict the progression or the prognosis of COVID-19 patients. Other implementations could be derived from the integration in our system of the “mild” and “severe” classes of COVID-19 images that could stratify the population according to disease severity or patient outcome (e.g., the need for mechanical ventilation, disease duration, and short-term survival). In order to develop these models, but also to improve our current model, it will be important to integrate biological and clinical data in the AI system.

Third, due to various reasons, the prevalence of bedside CXR was higher in the COVID-19 group than in negative cases. While this may appear as a potential source of bias, the use of bedside CXR does not underline more severe pneumonia, leading to the potentially incorrect association between higher severity and COVID-19. In fact, portable equipment allows for easier management and reduction of infections because they do not require infectious subjects to move around the hospital facilities and are easier to sanitize [56,57].

Finally, it is important to recognize that the role of CXR in evaluating patients depends on the severity of the infection in the individual patient and COVID-19 prevalence in the community [61,62]. In individuals who are asymptomatic, the sensitivity of CXR could fall steeply, in particular in the first 48 h after symptoms onset, since asymptomatic individuals could test positive at RT-PCR and negative at CXR. Moreover, CXR may prove less useful in areas with very little circulating SARS-CoV-2. Conversely, CXR is most useful in patients who are acutely ill and symptomatic in areas with relatively high prevalence. In this scenario, patients with CXR findings attributable to COVID-19 could be considered as presumptively infected by the virus when the first RT-PCR test result is still rarely negative. For the purpose of differential diagnosis, disease severity ought to be considered as a potential source of bias because COVID-19 pneumonia cases may potentially present as more severe, on average, than CAP.

## 5. Conclusions

In subjects with suspected COVID-19, an AI reader applied to CXR achieved a sensitivity ranging 0.80–0.98 and a specificity of 0.88 in the diagnosis of COVID-19, also attaining a sensitivity ranging 0.82–0.97 and a specificity ranging 0.82–0.96 in the differential diagnosis of COVID-19 versus CAP. This system may prove a cost-sustainable and efficient tool as a second opinion to radiologists in a variable spectrum of clinical and epidemiological contexts. It will be held in continuous training with new CXR images to increase its performance and provide a larger external validation.

## Figures and Tables

**Table 1:** Patients’ distribution among the included datasets.

	Timeframe	Center 1 Patients	Center 2 Patients	AIforCOVID Patients	Total
COVID-19	21 February to 16 March 2020	48	114	-	162
CAP	1 February to 16 March 2019	42	70	-	112
Negative		48	110	-	158
COVID-19	March to June 2020	-	-	820	820
Total	-	138	294	820	1252

COVID-19, patients with COVID-19 pneumonia; CAP, patients with community-acquired non-COVID-19 pneumonia; Negative, patients with negative chest X-ray exams.

**Table 2:** Training and validation (cross-validation) performance of the proposed AI system.

**COVID-19 Versus Negative**	**COVID-19 (n)**	**Negative** **(n)**
Assigned COVID-19	89	13
Assigned Negative	9	85
	Sensitivity 0.91	Specificity 0.87
**COVID-19 Versus CAP**	**COVID-19 (n)**	**CAP** **(n)**
Assigned COVID-19	83	16
Assigned CAP	15	72
	Sensitivity 0.85	Specificity 0.82

CAP, community-acquired non-COVID-19 pneumonia.

**Table 3:** Independent testing I performance of the proposed AI system.

**COVID-19 Versus Negative**	**COVID-19 (n)**	**Negative** **(n)**
Assigned COVID-19	63	7
Assigned Negative	1	53
	Sensitivity 0.98	Specificity 0.88
**COVID-19 Versus CAP**	**COVID-19 (n)**	**CAP** **(n)**
Assigned COVID-19	62	1
Assigned CAP	2	23
	Sensitivity 0.97	Specificity 0.96

CAP, community-acquired non-COVID-19 pneumonia.

**Table 4:** Independent testing II performance of the proposed AI system.

**COVID-19 Versus Negative**	**COVID-19 (n)**	**Negative** **(n)**
Assigned COVID-19	652	-
Assigned Negative	168	-
	Sensitivity 0.80	Specificity -
**COVID-19 Versus CAP**	**COVID-19 (n)**	**CAP** **(n)**
Assigned COVID-19	674	-
Assigned CAP	146	-
	Sensitivity 0.82	Specificity -

CAP, community-acquired non-COVID-19 pneumonia.

## Data Availability

Datasets from Center 1 (IRCCS Policlinico San Donato, San Donato Milanese, Italy) and Center 2 (ASST Monza—Ospedale San Gerardo, Monza, Italy) used and/or analyzed during the current study are available from the corresponding author on reasonable request. The AIforCOVID dataset is publicly available at https://aiforcovid.radiomica.it/ (accessed 14 March 2021).

## References

[1] Bordi L., Nicastri E., Scorzolini L., Di Caro A., Capobianchi M.R., Castilletti C., Lalle E. (2020). Differential diagnosis of illness in patients under investigation for the novel coronavirus (SARS-CoV-2), Italy, February 2020. Eurosurveillance.

[2] Lei P., Fan B., Wang P. (2020). Differential Diagnosis for Coronavirus Disease (COVID-19): Beyond Radiologic Features. Am. J. Roentgenol..

[3] Cheng M.P., Papenburg J., Desjardins M., Kanjilal S., Quach C., Libman M., Dittrich S., Yansouni C.P. (2020). Diagnostic Testing for Severe Acute Respiratory Syndrome-Related Coronavirus 2. Ann. Intern. Med..

[4] Jamil S., Mark N., Carlos G., Cruz C.S.D., Gross J.E., Pasnick S. (2020). Diagnosis and Management of COVID-19 Disease. Am. J. Respir. Crit. Care Med..

[5] Wynants L., van Calster B., Collins G.S., Riley R.D., Heinze G., Schuit E., Bonten M.M.J., Dahly D.L., Damen J.A., Debray T.P.A. (2020). Prediction models for diagnosis and prognosis of COVID-19: Systematic review and critical appraisal. BMJ.

[6] Yuen K.-S., Ye Z.-W., Fung S.-Y., Chan C.-P., Jin D.-Y. (2020). SARS-CoV-2 and COVID-19: The most important research questions. Cell Biosci..

[7] Franquet T., Jeong Y.J., Lam H.Y.S., Wong H.Y.F., Chang Y.-C., Chung M.J., Lee K.S. (2020). Imaging findings in coronavirus infections: SARS-CoV, MERS-CoV, and SARS-CoV-2. Br. J. Radiol..

[8] Hosseiny M., Kooraki S., Gholamrezanezhad A., Reddy S., Myers L. (2020). Radiology Perspective of Coronavirus Disease 2019 (COVID-19): Lessons from Severe Acute Respiratory Syndrome and Middle East Respiratory Syndrome. Am. J. Roentgenol..

[9] Waller J.V., Kaur P., Tucker A., Lin K.K., Diaz M.J., Henry T.S., Hope M. (2020). Diagnostic Tools for Coronavirus Disease (COVID-19): Comparing CT and RT-PCR Viral Nucleic Acid Testing. Am. J. Roentgenol..

[10] Akl E.A., Blažić I., Yaacoub S., Frija G., Chou R., Appiah J.A., Fatehi M., Flor N., Hitti E., Jafri H. (2021). Use of Chest Imaging in the Diagnosis and Management of COVID-19: A WHO Rapid Advice Guide. Radiology.

[11] Rubin G.D., Ryerson C.J., Haramati L.B., Sverzellati N., Kanne J.P., Raoof S., Schluger N.W., Volpi A., Yim J.-J., Martin I.B.K. (2020). The Role of Chest Imaging in Patient Management during the COVID-19 Pandemic: A Multinational Consensus Statement from the Fleischner Society. Radiology.

[12] Sverzellati N., Milanese G., Milone F., Balbi M., Ledda R.E., Silva M. (2020). Integrated Radiologic Algorithm for COVID-19 Pandemic. J. Thorac. Imaging.

[13] Kim H., Hong H., Yoon S.H.H. (2020). Diagnostic Performance of CT and Reverse Transcriptase Polymerase Chain Reaction for Coronavirus Disease 2019: A Meta-Analysis. Radiology.

[14] Mossa-Basha M., Medverd J., Linnau K.F., Lynch J.B., Wener M.H., Kicska G., Staiger T., Sahani D.V. (2020). Policies and Guidelines for COVID-19 Preparedness: Experiences from the University of Washington. Radiology.

[15] Mossa-Basha M., Meltzer C.C., Kim D.C., Tuite M.J., Kolli K.P., Tan B.S. (2020). Radiology Department Preparedness for COVID-19: Radiology Scientific Expert Review Panel. Radiology.

[16] Matos J., Paparo F., Mori M., Veneziano A., Sartini M., Cristina M.L., Rollandi G.A. (2020). Contamination inside CT gantry in the SARS-CoV-2 era. Eur. Radiol. Exp..

[17] Kwan K.E.L., Tan C.H. (2020). The humble chest radiograph: An overlooked diagnostic modality in the COVID-19 pandemic. Quant. Imaging Med. Surg..

[18] Koo H.J., Choi S.-H., Sung H., Choe J., Do K.-H. (2020). RadioGraphics Update: Radiographic and CT Features of Viral Pneumonia. RadioGraphics.

[19] Schiaffino S., Tritella S., Cozzi A., Carriero S., Blandi L., Ferraris L., Sardanelli F. (2020). Diagnostic Performance of Chest X-ray for COVID-19 Pneumonia During the SARS-CoV-2 Pandemic in Lombardy, Italy. J. Thorac. Imaging.

[20] Cozzi A., Schiaffino S., Arpaia F., Della Pepa G., Tritella S., Bertolotti P., Menicagli L., Monaco C.G., Carbonaro L.A., Spairani R. (2020). Chest X-ray in the COVID-19 pandemic: Radiologists’ real-world reader performance. Eur. J. Radiol..

[21] Wong H.Y.F., Lam H.Y.S., Fong A.H.-T., Leung S.T., Chin T.W.-Y., Lo C.S.Y., Lui M.M.-S., Lee J.C.Y., Chiu K.W.-H., Chung T.W.-H. (2020). Frequency and Distribution of Chest Radiographic Findings in Patients Positive for COVID-19. Radiology.

[22] Bernheim A., Mei X., Huang M., Yang Y., Fayad Z.A., Zhang N., Diao K., Lin B., Zhu X., Li K. (2020). Chest CT Findings in Coronavirus Disease-19 (COVID-19): Relationship to Duration of Infection. Radiology.

[23] RSNA Pneumonia Detection Challenge 2018. https://www.rsna.org/education/ai-resources-and-training/ai-image-challenge/rsna-pneumonia-detection-challenge-2018.

[24] Chouhan V., Singh S.K., Khamparia A., Gupta D., Tiwari P., Moreira C., Damaševičius R., de Albuquerque V.H.C. (2020). A Novel Transfer Learning Based Approach for Pneumonia Detection in Chest X-ray Images. Appl. Sci..

[25] Fischer A.M., Yacoub B., Savage R.H., Martinez J.D., Wichmann J.L., Sahbaee P., Grbic S., Varga-Szemes A., Schoepf U.J. (2020). Machine Learning/Deep Neuronal Network. J. Thorac. Imaging.

[26] Kana E.B.G., Kana M.G.Z., Kana A.F.D., Kenfack R.H.A. (2020). A Web-based Diagnostic Tool for COVID-19 Using Machine Learning on Chest Radiographs (CXR). medRxiv.

[27] Barstugan M., Ozkaya U., Ozturk S. (2020). Coronavirus (COVID-19) Classification Using CT Images by Machine Learning Methods. arXiv.

[28] Elaziz M.A., Hosny K.M., Salah A., Darwish M.M., Lu S., Sahlol A.T. (2020). New machine learning method for image-based diagnosis of COVID-19. PLoS ONE.

[29] Li L., Qin L., Xu Z., Yin Y., Wang X., Kong B., Bai J., Lu Y., Fang Z., Song Q. (2020). Using Artificial Intelligence to Detect COVID-19 and Community-acquired Pneumonia Based on Pulmonary CT: Evaluation of the Diagnostic Accuracy. Radiology.

[30] Hurt B., Kligerman S., Hsiao A. (2020). Deep Learning Localization of Pneumonia. J. Thorac. Imaging.

[31] Pereira R.M., Bertolini D., Teixeira L.O., Silla C.N., Costa Y.M.G. (2020). COVID-19 identification in chest X-ray images on flat and hierarchical classification scenarios. Comput. Methods Programs Biomed..

[32] Apostolopoulos I.D., Aznaouridis S.I., Tzani M.A. (2020). Extracting Possibly Representative COVID-19 Biomarkers from X-ray Images with Deep Learning Approach and Image Data Related to Pulmonary Diseases. J. Med. Biol. Eng..

[33] Amyar A., Modzelewski R., Li H., Ruan S. (2020). Multi-task deep learning based CT imaging analysis for COVID-19 pneumonia: Classification and segmentation. Comput. Biol. Med..

[34] Kumar R., Khan A.A., Zhang S., Kumar J., Yang T., Golalirz N.A., Ali I., Shafiq S., Wang W. (2020). Blockchain-Federated-Learning and Deep Learning Models for COVID-19 Detection Using CT Imaging. arXiv.

[35] Zhang H., Zhang J., Zhang H., Nan Y., Zhao Y., Fu E., Xie Y., Liu W., Li W., Zhang H. (2020). Automated detection and quantification of COVID-19 pneumonia: CT imaging analysis by a deep learning-based software. Eur. J. Nucl. Med. Mol. Imaging.

[36] Castiglioni I., Ippolito D., Interlenghi M., Monti C.B., Salvatore C., Schiaffino S., Polidori A., Gandola D., Messa C., Sardanelli F. (2021). Machine learning applied on chest X-ray can aid in the diagnosis of COVID-19: A first experience from Lombardy, Italy. Eur. Radiol. Exp..

[37] Khuzani A.Z., Heidari M., Shariati S.A. (2020). COVID-Classifier: An automated machine learning model to assist in the diagnosis of COVID-19 infection in chest X-ray images. medRxiv.

[38] Minaee S., Kafieh R., Sonka M., Yazdani S., Soufi G.J. (2020). Deep-COVID: Predicting COVID-19 from chest X-ray images using deep transfer learning. Med. Image Anal..

[39] Rahaman M.M., Li C., Yao Y., Kulwa F., Rahman M.A., Wang Q., Qi S., Kong F., Zhu X., Zhao X. (2020). Identification of COVID-19 samples from chest X-ray images using deep learning: A comparison of transfer learning approaches. J. Xray Sci. Technol..

[40] Ucar F., Korkmaz D. (2020). COVIDiagnosis-Net: Deep Bayes-SqueezeNet based diagnosis of the coronavirus disease 2019 (COVID-19) from X-ray images. Med. Hypotheses.

[41] Farhat H., Sakr G.E., Kilany R. (2020). Deep learning applications in pulmonary medical imaging: Recent updates and insights on COVID-19. Mach. Vis. Appl..

[42] Shi F., Wang J., Shi J., Wu Z., Wang Q., Tang Z., He K., Shi Y., Shen D. (2020). Review of Artificial Intelligence Techniques in Imaging Data Acquisition, Segmentation and Diagnosis for COVID-19. IEEE Rev. Biomed. Eng..

[43] López-Cabrera J.D., Orozco-Morales R., Portal-Diaz J.A., Lovelle-Enríquez O., Pérez-Díaz M. (2021). Current limitations to identify COVID-19 using artificial intelligence with chest X-ray imaging. Health Technol..

[44] Castiglioni I., Rundo L., Codari M., Di Leo G., Salvatore C., Interlenghi M., Gallivanone F., Cozzi A., D’Amico N.C., Sardanelli F. (2021). AI applications to medical images: From machine learning to deep learning. Phys. Med..

[45] Soda P., D’Amico N.C., Tessadori J., Valbusa G., Guarrasi V., Bortolotto C., Akbar M.U., Sicilia R., Cordelli E., Fazzini D. (2020). AIforCOVID: Predicting the Clinical Outcomes in Patients with COVID-19 Applying AI to Chest-X-rays. An Italian Multicentre Study. arXiv.

[46] TRACE4. http://www.deeptracetech.com/temp/TechnicalSheet__TRACE4.pdf.

[47] Zwanenburg A., Vallières M., Abdalah M.A., Aerts H.J.W.L., Andrearczyk V., Apte A., Ashrafinia S., Bakas S., Beukinga R.J., Boellaard R. (2020). The Image Biomarker Standardization Initiative: Standardized Quantitative Radiomics for High-Throughput Image-based Phenotyping. Radiology.

[48] He K., Zhang X., Ren S., Sun J. (2015). Deep Residual Learning for Image Recognition. arXiv.

[49] Gaudart J., Landier J., Huiart L., Legendre E., Lehot L., Bendiane M.K., Chiche L., Petitjean A., Mosnier E., Kirakoya-Samadoulougou F. (2021). Factors associated with the spatial heterogeneity of the first wave of COVID-19 in France: A nationwide geo-epidemiological study. Lancet Public Health.

[50] Van Damme W., Dahake R., Delamou A., Ingelbeen B., Wouters E., Vanham G., van de Pas R., Dossou J.-P., Ir P., Abimbola S. (2020). The COVID-19 pandemic: Diverse contexts; different epidemics—how and why?. BMJ Glob. Health.

[51] Quilty B.J., Clifford S., Hellewell J., Russell T.W., Kucharski A.J., Flasche S., Edmunds W.J., Atkins K.E., Foss A.M., Waterlow N.R. (2021). Quarantine and testing strategies in contact tracing for SARS-CoV-2: A modelling study. Lancet Public Health.

[52] Clark A., Jit M., Warren-Gash C., Guthrie B., Wang H.H.X., Mercer S.W., Sanderson C., McKee M., Troeger C., Ong K.L. (2020). Global, regional, and national estimates of the population at increased risk of severe COVID-19 due to underlying health conditions in 2020: A modelling study. Lancet Glob. Health.

[53] Lauring A.S., Hodcroft E.B. (2021). Genetic Variants of SARS-CoV-2—What Do They Mean?. JAMA.

[54] Mascola J.R., Graham B.S., Fauci A.S. (2021). SARS-CoV-2 Viral Variants-Tackling a Moving Target. JAMA.

[55] Moore J.P., Offit P.A. (2021). SARS-CoV-2 Vaccines and the Growing Threat of Viral Variants. JAMA.

[56] Zanardo M., Schiaffino S., Sardanelli F. (2020). Bringing radiology to patient’s home using mobile equipment: A weapon to fight COVID-19 pandemic. Clin. Imaging.

[57] Wu G., Li X. (2020). Mobile X-rays are highly valuable for critically ill COVID patients. Eur. Radiol..

[58] Franquet T. (2018). Imaging of Community-acquired Pneumonia. J. Thorac. Imaging.

[59] Koo H.J., Lim S., Choe J., Choi S.-H., Sung H., Do K.-H. (2018). Radiographic and CT Features of Viral Pneumonia. RadioGraphics.

[60] Franquet T. (2011). Imaging of Pulmonary Viral Pneumonia. Radiology.

[61] Sardanelli F., Di Leo G. (2020). Assessing the Value of Diagnostic Tests in the Coronavirus Disease 2019 Pandemic. Radiology.

[62] Leeflang M.M.G., Bossuyt P.M.M., Irwig L. (2009). Diagnostic test accuracy may vary with prevalence: Implications for evidence-based diagnosis. J. Clin. Epidemiol..

[63] Eltorai A.E.M., Bratt A.K., Guo H.H. (2020). Thoracic Radiologists’ Versus Computer Scientists’ Perspectives on the Future of Artificial Intelligence in Radiology. J. Thorac. Imaging.

